# Book Reviews

**Published:** 1889-01

**Authors:** 


### Book Reviews.

---

**Treatise on the Diseases of Women.** For the use of students and practitioners. By ALEXANDER J. C. SKENE, M. D., Professor of Gynecology in the Long Island College Hospital, Brooklyn. With 251 engravings and nine chromo-lithographs. 8vo, pp. xiv., 960. New York: D. Appleton & Co. 1888.

As this is the most important American contribution to the text-book literature of Gynecology that has appeared since Dr. Emmet's work was published (1879), it demands an extended notice.

When an author has already given to the profession a work which is regarded as classic, a forthcoming volume from his hand is looked for with more than an ordinary degree of expectancy. Dr. Skene's treatise on the Diseases of the Bladder and Urethra in Women needs no word of praise here. Embodied as it is in the present work, it is hoped that should any one have failed to possess it, the fact of its presence in company with the present volume will be dwelt upon by the diligent agent as a favorable opportunity for securing a volume, his ignorance of which will be the more regretted the better he becomes acquainted with it.

It is now our purpose to notice the remaining portion of the volume. Regarding the book as a work for "students and practitioners," we cannot but feel that the intentional omission of all bibliography will detract more than a little from its value. Occupying the position in didactic medicine that he does, it would be of more than passing interest and value to both student and practitioner to know the lines Dr. Skene has followed, the authors he has consulted, and the opinions he has deemed worthy of implicit confidence; all the more that in these days of gynecological frenzy so much is perpetrated upon the reading profession, that it is good to know an honest author's opinion of what is good to avoid.

The introduction into the book of cases, making the volume to some extent clinical, is of evident advantage to the student, though it will be somewhat tedious to the practical surgeon. The illustrations are, in the main, excellent, and add much to the perspicacity of the text. Nowhere more than in the selection and abridgment of cuts has the author used a better judgment. Simplicity of design, with only enough to explain a condition, is infinitely better than an agony of elaboration, driving the reader to a degree of distraction, only equaled by his earlier wrestlings with the complexities of analytical geometry and conic sections.

Of the subject-matter of the book a fair estimate can only be had by examining it in detail. The opening chapter upon "Methods of Examination" will repay the careful reading of the learner. In the manner of making the bi-manual examination, however, we do not agree with the author. Two fingers are infinitely better than one introduced into the vagina. The rectal touch also is especially to be advised in the unmarried, and is of use is *every* examination that would be considered thorough. In a work designed for the use of students, we cannot but feel that the author regards the use of the uterine sound with by far too much complacency. With students, and the unpracticed or unskilled, this instrument may be necessary for diagnosis; yet these are the very persons likely to do the most harm with it. Careful bi-manual examination will in most cases give exact information as to the size, situation, and mobility of the uterus, without recourse to an instrument whose existence is its only excuse for mention in a work upon gynecology. In all cases, an examination properly made will give information *sufficiently exact* for diagnosis and treatment without the sound.

Passing over the chapter upon the development of the sexual organs, which is concise and clear, as is also the chapter upon the arrest of development, we come to the consideration of flexions of the uterus. Here the author's discussion is at once lucid and logical. His view that flexions of the uterus are congenital, caused by arrest of development during the latter stage of that process, is warranted, we believe, by a careful consideration of all facts. That they are also due to the changes occurring during the secondary development of puberty, is also a statement admitting but little question. This, of course, cannot apply to those cases of retroflexion occurring after delivery, where a heavy uterus falls by its own weight into any of the vicious positions. The various flexions are well depicted. From what we have said of the uterine sound, we cannot concur in its use as an agent for the correction of the position of an abnormally placed uterus. Where invagination of the organ is imperfect, the operation suggested will doubtless be of utility; but, where reposition of the organ is required, that, it seems to us, can be accomplished by judicious packing, meanwhile applying the uterine repositor to the fundus, through the vaginal wall, with the patient in the knee-chest position. The use of the stem pessary for the correction of this deformity is to be deprecated. Its use we think is only to be allowed where the most careful observation is continually had of the patient, and then only with the greatest care. All in all, we believe the stem pessary capable of doing more harm than good, and hence to be avoided. When Dr. Skene says: "It should be borne in mind that there is a tendency for the flexion and all consequent symptoms to return unless utero-gestation follows," we believe his experience is rather to be followed than his suggestion to use an instrument, which, while dangerous, does not afford any assurance of success, in the condition for which it is applied.

The chapter upon the diseases and malformations of the vagina, leaves nothing to be desired. In the treatment of specific vaginitis, in view of the dangers of its transmission to the uterus and thence to the Fallopian tubes and ovaries, possibly a little more stress upon the importance of its early recognition and proper treatment, even to severity, would not have been out of place.

Chapter VII., in which Injuries to the Pelvic Floor are discussed, is one of the most readable portions of the volume. The anatomical relations of the perineal muscles, relative to lacerated perineum, is discussed most rationally. The function of the perineal muscles is thus concisely stated: "When pressure is made downward by any body in the rectum or vagina, the perineal muscle does not draw the orifices of these canals upwards, and hence supply a resisting force to the downward pressure, which effects dilatation of the vagina or rectum." Dr. Skene believes that the perineal body supports the pelvic organs, and that in only the three following conditions expected prolapse of the pelvic organs will follow its injury:

"1. When the injury is compensated for by the muscles (which still maintain their attachment to the vagina and rectum) drawing the remaining portion of the pelvic floor upward, forward, and toward the pubes, thereby closing the vaginal orifice and supporting the pelvic organs.

"2. Where, by reason of some intra-pelvic inflammation, the organs have become fixed by adhesion; and,

"3. Where the patient is abundantly supplied with adipose tissue, and takes very little active exercise."

The subcutaneous laceration of the muscles of the perineum is intelligently discussed. The author considers the conditions too frequently mistaken for functional imperfection of the perineum, or relaxation. It is evident that this latter term is a misnomer, the laceration being just as real as where the tear extends entirely through the sphincter. A form of injury specially dwelt upon is injury to the levator ani muscles, without implication of the other perineal muscles. In this condition, "The whole pelvic floor, including the rectum, vagina, and lower part of the labia, projects downward below its normal elevation. This suggests the thought that subcutaneous laceration of the transversus perinei takes place also when the levator ani is injured."

Nothing further need be noticed here save the directions for operation in the various kinds and degrees of laceration. These are clear, and the illustrations are excellent, even superior, all in all, to any others we have seen, in plainly indicating the steps of the operation they are meant to explain.

There is a *conspicuous* absence of the name of Dr. Emmet, as related to the operation for restoring the perineum by the now universally recognized "*Emmet's Operation.*"

The directions for the after-care of the patient, subsequent to perineal or vaginal operation, are wise, and if followed will prevent much useless meddling as senseless as harmful.

Of the chapter on Fistula in Ano, little need be said. The subject is fully considered in all surgical treatises, and deserves no special mention, save to say that the so-called "new" operation of laying open the fistula, paring the edges and suturing them is, we think, not *properly accredited.* Like all other operations, it is liable to fail. This liability increases where the fistula is deep and an abundance of adipose tissue present, and where the vitality of the patient is low.

In the treatment of inflammatory affections of the uterus, while not rejecting all intra-uterine medication, Dr. Skene is to a marked degree conservative. He says: "Bearing in mind that the uterus should not be injured in its structure, the therapeutist is bound to reject all the more powerful and destructive agents, such as nitric or chromic acid, caustic potash, and the actual cautery." Further on, however, in the treatment of uterine fibroids, in case the cervix cannot be reached by the electrode, he admits the puncture of the organ by a needle-pointed electrode, and says the current must be strong enough to destroy the tissues in immediate contact with it. Here is an apparent inconsistency, into which the enthusiasm of the electro-maniacs has apparently betrayed the conservatism of the author. The matter will again be referred to further on.

Referring to the agents above enumerated as dangerous, he continues: "Leaving out of account the value of these potent agents in the treatment of malignant diseases of the uterus, I desire to be distinctly understood as opposed to their use in the treatment of benign uterine diseases. . . . . . . . Nitric and chromic acids and other caustics, are being laid aside, but only, I fear, to be given place in some cases to new but none the less destructive agents. I allude to the galvano-cautery and thermo-cautery." In benign diseases of the uterus, the author sanctions the use of chloride of zinc, in one or two grains to the ounce, for internal application. We think the farther chloride of zinc is from the gynecological table, the happier and safer will be the patient.

In the treatment of corporeal endometritis, the key-note of the author's practice is again sounded: "THERE IS SO MUCH RISK IN TREATING THE MUCOUS MEMBRANE OF THE CAVITY OF THE UTERUS, THAT THERE ARE CERTAIN PRECAUTIONS WHICH SHOULD BE KEPT IN MIND. . . . . . Intra-uterine applications exciting to the cervical canal should not be used until other means have been thoroughly tried and have failed. The uterus should be in or near its normal position. The cervix uteri should be sufficiently dilated to allow any excess of the fluid to escape from the cavity of the body." In view of these directions the facts stand boldly out, that a therapeutic measure of such danger should not be advocated for the many, but only for the expert, if at all. An operation over which it is necessary to write "caution," as over poison-vials in the drug shop, had better be kept out of the general reach. The operation of scraping the uterus, at present so much in vogue, often to the detriment of the patient, by serving to light up acute inflammations, likely to extend to the tubes and ovaries, Dr. Skene advises always to be done with the blunt curette. The counsel is good, and with care will reduce the danger of the operation to a minimum.

The treatment of subinvolution is next considered. It seems strange that a practitioner of such wide experience as Dr. Skene should have never observed a case subsequent to parturition, the diagnosis of which he could be sure. We do not believe the condition so rare as this view would seem to make it. We concur in the writer's opinion, that in such conditions, the use of electricity, by reason of its stimulating effects upon the uterine muscle, promises an efficient and harmless remedy. Concerning sclerosis of the uterus, and membranous dysmenorrhea, little need be said. When the author dismisses the prognosis of the first with the assertion that recovery with or without treatment is the exception, and ends his discussion of the latter, remarking that several other cases might be added, some showing failure of treatment, and others where the patients were really made worse by being treated for inflammation of the uterus, which was supposed to be the cause of the affection, but undoubtedly was not, and that other cases might be given, also in which recovery took place, and that after several months or years the trouble returned, there would seem to be little use in multiplying words, especially concerning the latter affection, of whose pathology little is known, and of its treatment still less.

Chapter XIV., treating of lacerations of the cervix from parturition, gives an excellent discussion of this prevalent lesion. There is, however, little to be said concerning the operation, as it has already been discussed as the brilliant achievement of Dr. Emmet. Those who do not know the procedure devised by Dr. Emmet, can nowhere get a more concise statement of the method than is here enunciated. Added to an excellent description are admirable plates. The two combined leave nothing to be added. It may be well, however, to notice the danger of the operation. Before it is performed it is *better in every* instance to determine absolutely whether there is *pre-existing* tubal or ovarian trouble. If so, it will in all probability be lighted up anew by the surgical interference lower down. The same is to be feared, under similar circumstances, of the perineal operation. To the chapter on inversion of the uterus, we can only refer in passing. The reader here, as elsewhere, will find full illustrations of the subject. He will likely find but little use for the subject-matter, as the condition is fortunately comparatively rare. The one brief reference to the late Dr. James P. White, who was a pioneer in this field, does but slight justice to his achievements in reducing the inverted uterus.

Of the displacements of the uterus, our author considers the backward and downward varieties only to demand discussion. Prolapsus, with all its attendant evils, is fully considered, as is also its causation. Of the surgical interferences for the condition, he says: "I find (comparing his results with Martin's) I have succeeded as well by the continued use of mechanical supports and surgical operations. That in the treatment of prolapsus, where operation upon the cervix uteri and pelvic floor has failed, kolporrhaphy has also been useless. I have, therefore, abandoned that operation." Dr. Skene's verdict is against the vaginal stem pessary in this condition. By a careful use of the ring, tampon, and T bandage, he believes he can accomplish all that can be done by the more elaborate supports.

The argument of retroversion is lucidly stated, but here again we must interpose our earnest protest against the use of the sound as a replacing instrument. It is not needed, and should not be used. The support given by pessaries is explained together, where and how they at times will do harm, and fail of good. Prolapse of the ovaries, especially if adherent, is noted as a frequent negation of success.

The remedy here, if the condition can be at all obviated, is persistent packing in the knee-chest position, with constant well-directed efforts to replace the truant organs by the use of a rubber repositor. This form of repositor we think preferable to the sponge suggested by the author. The explanation of the mechanics of the pessary is a feature well worth the perusal of the student, and also of many who for a long time have been applying the instrument. Of the Alexander operation, for shortening the round ligament in this condition, Dr. Skene is inclined to believe its value still undetermined. He here differs from most authorities, but his conservatism seems in part justified by some aspects of the condition. At this point, two cases of Prof. W. M. Polk are introduced as illustrative of the subject. They by far had better been omitted. They teach nothing, are simply surgical curiosities, and illustrate how a desire for novelty can subvert common sense even in surgery. The chapter on the abuse of pessaries must be read, and its illustrations studied, to be appreciated. Those of us who have ever recklessly introduced a pessary, as the ancient farriers made the horse's hoof fit their shoes by paring down the hoof, must have pangs of conscience as the suffering caused thereby is contemplated. Misplacement of any of the forms of the pessary causes excruciating pain, varying somewhat as to the malposition. The pain is, however, the salvation of the patient, who, because she cannot endure it, demands the withdrawal of the offending body. This is not so true of the ring form, which, if too large, frequently ulcerates through the vagina into the rectum.

In dealing with the hypertrophy of the cervix uteri, Dr. Skene disregards the refinements of the German writers, and, without wasting time with names, treats the enlargement as he finds it, to-wit, as a simple hypertrophy to be gotten rid of. His use of the rubber cord, though rarely tried in this operation, is, we are convinced, wise. We again cannot refrain from referring here, as we so often have done, to the excellent illustration of the operation. With it before him, the veriest dullard must understand the method of amputating the cervix.

We now come to a subject much discussed at the present time, Fibroid Tumors of the Uterus. Drawing special attention to the form of fibroma growing within the folds of the broad ligament, as hitherto but little described, Dr. Skene goes on to discuss the various forms and appearances of the disease much as we have already learned them. His observation that these tumors sometimes soften and break down, is correct. One such case with like termination has fallen under the reviewer's notice. Of medicinal agents, ergot is regarded as the most effective in controlling hemorrhage, diminishing the blood supply of the tumor, and thereby retarding its growth; sometimes, also, exciting the expulsion of the neoplasm.

For mechanically removing a pedunculated tumor, the author prefers the wire écraseur. The removal by this means of the tumors, before they have become pedunculated, is held to be dangerous. Removal of the tubes and ovaries in order to retard or control the growth of these tumors, the author regards as the best treatment in properly selected cases. It is a matter worthy of comment that reliable observers have found that in fibroid disease of the uterus, the ovaries and tubes are generally found diseased. The fact being accepted, it would appear that the removal of the appendages in this condition is a logical procedure, even if the tumor is pedunculated. We have already referred to the treatment of these tumors by electro-puncture. Electricity, in its effect upon any disease, is as mysterious as the agent itself. Its existence as a force of nature is indisputable, yet the raison d'étre of its existence is perhaps as much unsolved as ever. Philosophers explain it, and the ignorant play with it. The methods of its application in disease are as various as the ages of the operators. The effects claimed for it are as diverse as the enthusiasm of converts, whose limit in the application of the current finds fit expression in "Lay on, Macduff, and damned be he that first cries, 'Hold, enough!'" and the doubts of sceptics, who do not use it at all. Somewhere between the two will eventually be found the differential of its value. Its applications range between the wearing of a toy belt and the passing of currents, sufficient to light an incandescent burner, through the "too, too solid flesh," and this "must give us pause." To pass a current into a tumor, either by electrolysis that will dissolve it perceptibly, or by the galvano-cautery that will destroy it by burning, is in the one case to toy with a current which, if it is a "short circuit," must kill the patient in the first, and in the second, subject her to all the dangers that a goad, heated by any other means than the mysterious electric current, would create. We believe that small tumors may be influenced in their growth and nutrition by an electrolytic current, because here a strength of current may be applied not incompatible with safety, because of the smaller resistance through a smaller growth. The converse of this proposition negatives the successful application in tumors of a larger growth. As to the application of these currents, tentative as they must be, because as yet undetermined, we maintain they should be applied not by men whose enthusiasm is far in excess of their knowledge, and whose temerity is vast because their judgment is little, but by men accustomed to handle this subtle agent, whose experience admonishes them where safety ends, where danger begins. Dr. Skene quotes a number of cases illustrative of fibroid disease of the uterus from Mr. Keith's "Contributions to the Surgical Treatment of Tumors of the Abdomen." To those who are unfamiliar with the little though great book, the citations will be a revelation as to the possibilities of abdominal surgery at the hands of a man at once conservative, skilful and bold.

To those who, with an admiration almost amounting to reverence, have studied the book, loving the author for his childlike simplicity, and absence of parading egotism in the light of his wonderful successes, though a pioneer in the work his genius illumines — his apostasy from the fast and firm principles governing the treatment of these tumors, laid down by himself, to the empirical treatment of electricity, is a disappointment keen and bitter.

Of the treatment of malignant diseases of the uterus, no extended mention is required. Hysterectomy, as a remedy for cancer of the cervix, is yet an open question. Again, whether removal of the entire organ, or an extended removal in the lines of amputation of the cervix, is to be preferred, is still a question sub judice. As the author remarks, hysterectomy is obviously the only operation for cancer of the body of the uterus.

From the chapter on the menopause we shall only make two quotations without comment: "Any disease or derangement of the functions of the sexual organs which may exist when the patient is drawing near to the time for the cessation of the menses, should be attended to. Much harm has arisen by physicians advising patients who are suffering from symptoms referring to the pelvic organs, to have patience, and they will be all right after the change comes." "A long list of diseases has been given as occurring at the menopause. The list covers nearly all the diseases that flesh is heir to. The majority of these have no relations to the menopause excepting that when there is a predisposition to any disease, the disturbances of the system due to the change, would favor the outbreak at that time." The chapters on the diseases of the ovaries, Fallopian tubes, pelvic cellulitis, peritonitis, and hematocele, are the last to which we shall refer.

The chapters on diseases of the ovaries, tubes, and ovariotomy are at once interesting and instructive, in that they acknowledge fully the frequent *gonorrheal origin* of ovarian and tubal disease, and appreciate the value of *drainage* in abdominal surgery, and in other respects are scientific and sensible throughout.

Concerning the effect of removal of the tubes and ovaries on the female organism, especially in reference to the sexual desire, we believe Dr. Skene correct in his belief that no fixed rule can be laid down. In our own experience, different operations have had different after-effects. Whether, as time progresses, the variations will converge, bringing the results to something approaching unanimity, a carefully made statistical analysis alone can decide. In chronic ovaritis, where removal is decided upon, Dr. Skene believes both organs should be taken away; or rather that there "is less likelihood of erring in removing both."

Prolapse of the ovaries is recognized as one of the vexatious and often unmanageable affections of these little bodies. If the organs cannot be replaced by position, and pressure made in the vagina judiciously applied, removal is to be the resort, unless the patient is near the climacteric. The advice is in some respects questionable. We incline to the opinion, that in every case the final treatment is to be regulated by the symptoms and pain, irrespective of age. In younger patients, where pregnancy is possible, this might overcome adhesions otherwise not amenable to cure save by operation. If the adhesions are so organized that the uterus is firmly fixed, nothing, of course, save operation will be of benefit.

Of the various ovarian neoplasms no lengthy mention is here demanded. In the diagnosis of the simple ovarian cyst, the writer still accords some importance to Drysdale's ovarian corpuscle, an opinion in which few will agree with him. If any positive diagnosis of these growths is possible, a great step will have been taken when it is discovered. We do not believe such a stage has been reached. Of the early stage in the development of these tumors, Dr. Skene agrees with other authorities that there is nothing diagnostic. Of the succeeding stages, a differential table of diagnosis is given from Peaslee, a valuable addition to the volume. Where there is a doubt as to whether the tumor is a pregnancy or an ovarian tumor, it is advised to delay operation until the end of the time of gestation. Early operation in such a case is thus rebuked: "The fact is that those who are the least capable of making a diagnosis are the most inclined to operate early, and this I presume accounts for the mistakes recorded." The criticism we believe is more pungent than just. More tumors by far are diagnosed to be pregnancies, than pregnancies are mistaken for tumors. Further, a period of some months may make a vital difference in the nature of a tumor, its adhesions, etc., while in case of doubt, in skilful hands, an exploratory incision is free from danger, and is justifiable. Dr. Bantock long ago presented unanswerable argument on this line in his "Plea for Early Ovariotomy." Where there is suspicion that a tumor may be due to extra-uterine pregnancy, Dr. Skene suggests the application of electricity. On this point our convictions are antipodal. If there is any suspicion in surgery that justifies an exploratory incision, it is that of extra-uterine pregnancy in the earliest months. We regret that a chapter on this interesting and much disputed subject is not included in the book.

In the matter of the statistics of the operation, Dr. Skene is surprisingly at sea. Thackeray relates how in traveling in Scotland he came upon a sign at an inn, with the inscription, "The Battle of———," and on it was one Scotchman with a broad-sword. The citation of Keith's once surprisingly low mortality at this time possesses a näivete not exceeded by the Scotchman's idea of ———. This with all honor to Mr. Keith. The general considerations of ovariotomy should be read by all surgeons, by all students, and by all practitioners, many of whom, if the reports in the journals are to be trusted, regard opening the abdomen as a pleasant novelty, to be enjoyed the first time the occasion offered itself,—for "fools rush in, where angels fear to tread."

Concerning drainage, Dr. Skene says: "I believe that I can see that those who employ drainage have the best of it." We are happy to find so positive a committal to this principle, and cannot too strongly insist upon its correctness. As to the use of chemical antisepsis in this operation, we do not for a moment believe that it is necessary, or advisable, or safe. On this point, Keith, who says of the carbolic spray, "I have expected much and have got nothing," might again have been quoted. And again: "If one be careful enough—and a few are careful enough—one may do all this with boiled water alone." Of the diseases of the Fallopian tubes, the author has comparatively little to say. The diagnosis, symptomatology, prognosis, and treatment are briefly given, the last in every case being removal. Illustrative cases are cited.

Pelvic cellulitis and peritonitis call for attention in this notice only for the sake of insisting upon the strong probability that their causation is due to an external infection—puerperal sepsis, abortion, unclean instruments, gonorrhea, and the like. From this standpoint, pelvic cellulitis is secondary to pelvic peritonitis. That a cellulitis may not originate by direct injury to the cellular tissue is not denied, or that it may not be of lymphatic origin is not questioned. Our belief is, nevertheless, that, in a great majority of cases, it is due to external infection. In these cases, free incision and drainage are to be advocated.

Pelvic hematocele, its causation and treatment, remains to be briefly referred to. The divisions retained by the author are sub- and intra-peritoneal hematocele. The ordinary sources of the intra-peritoneal variety, that which deserves the most attention, are enumerated without including that due to a rupture of old adhesions between the pelvic peritoneum and displaced tubes and ovaries. The production of this lesion is, perhaps, the greatest danger of the so-called massage attempts to break up these adhesions. Where hematocele exists, instead of the expectant treatment, we cannot but think that surgical interference will do much to obviate its dangers, and give, all in all, a better chance of success to the patient. The principal cause of both varieties is, undoubtedly, tubal pregnancy.

In closing this review, we congratulate Dr. Skene upon the merit of his book. The points on which we have differed from him are legitimate questions of discussion, and do not prevent a full recognition of the worth of the book, as valuable to both "Students and Practitioners."

The publishers, as is their custom, have made a most beautiful and attractive volume.

---

**Wood's Medical and Surgical Monographs.** Volume I., number 1, containing The Pedigree of Disease, by JONATHAN HUTCHINSON, F. R. S.; Common Diseases of the Skin, by ROBERT M. SIMON, M. D.; and Varieties and Treatment of Bronchitis, by DR. FERRAND. 8vo, pp. 259. New York: William Wood & Co. 1888.

With their usual enterprise to furnish first-class literature in a form and for a price in reach of every physician, Wm. Wood & Co. announce the publication, in a series of monthly volumes, of monographs upon medical and surgical subjects. The first of these monographs is noticed above. It is bound in heavy paper; the press-work and paper are all that could be desired; the type is clear, and in every way the workmanship in getting up the volume is to be commended. So much for the mechanical part of the publishers' work.

The contents of the first volume requires more extended treatment. There can be no question to the opinion that the three articles that make up the first issue should be read and studied by every physician. The one on "The Pedigree of Disease," by Mr. Jonathan Hutchinson, opens up a subject of vast importance. How can one know and understand disease unless he is familiar with its pedigree? We recall that Dr. Holmes has somewhere said, that if he had been called a few generations before, he could have successfully treated a certain case. It was to compensate for the impossibility of complying with a wish, however commendable, like Dr. Holmes's, that these lectures were undertaken. They are six in number, and divide the subject under the heads of Temperament, Idiosyncrasy, and Diathesis. No abstract would do justice to these lectures, and we must content ourselves with giving the author's definition of these terms. *Temperament* is "applicable to the sum of the physical peculiarities of the individual, exclusive of all definite tendencies to disease." It does not imply disease, or even a tendency thereto, and is compatible with enjoyment of perfect health. *Diathesis* is any bodily condition, however induced, in virtue of which the individual is prone to suffer from some peculiar type of disease. These conditions may be inherited or acquired, and may be permanent, transitory, or recurrent. *Idiosyncrasy* is applicable to any definite peculiarity of organization, of which the consequences may occur unexpectedly and otherwise inexplicably. It does not imply any proneness to disease, but indicates that under certain conditions results peculiar to the individual will occur.

The second article is a series of lectures on the "Common Diseases of the Skin," by Dr. Robert M. Simon, of Birmingham, England, and is reproduced in this country for the first time. The common diseases are eczema, psoriasis, scabies, acne, and ringworm. There is also an introductory lecture on pruritus, a condition common to many of the diseases of the skin, and to nearly all of them seen in general practice. These lectures are clear and to the point. The subjects of which they treat are not made obscure by too great refinement, and it will certainly repay anyone to study them carefully.

The last article is by Dr. Ferrand, and consists of lectures upon "The Varieties and the Treatment of Bronchitis." This is the first time these lectures have appeared in English. They are fourteen in number, and divide bronchitis into the following varieties: Congestive, catarrhal, inflammatory, spasmodic, and infectious. Each variety is discussed as to its etiology, symptomatology, and treatment. The style in which these lectures are written is most attractive, and lends a charm that holds one until they are finished. They are comprehensive. For instance, in speaking of the varieties of spasmodic bronchitis, or asthma, the author first refers to the *asthma of the nose*, or the so-called *hay-fever*. He holds that the latter disease is thus central in its origin, or at least partially so, and this coincides with the opinion of many writers upon this subject. That hay-fever is something else than a nasal disease, accounts often for the lack of success in its treatment, when the therapeutics are directed entirely to that organ. For other instances we must refer the reader to the lectures themselves.

It is proper to state that the subscription to these monographs is ten dollars a year. For single copies the price is one dollar.

The next issue is announced to contain: "Gonorrheal Infection in Women," by William J. Sinclair, M. A., M. D.; "On Giddiness," by Thomas G. Stewart, M. D., etc., Professor of Physic and Clinical Medicine, University of Edinburgh; and "A Critical Study of the Clinical Value of Albuminuria in Bright's Disease," by Dr. Pierre Jeanton, of Paris.

---

**Medical Diagnosis—A Manual of Clinical Methods.** By J. GRAHAM BROWN, M. D., Fellow of the Royal College of Physicians, Edinburgh, etc. Second edition; illustrated. 8vo, pp. 288. New York: E. B. Treat, 771 Broadway. 1888.

This volume is one of the well-known series of "Medical Classics," and, as we think, is one of the most useful numbers that the publisher has issued in this form. The arrangement of the order of subjects is original, and based upon the physiological systems of the body, viz.: Alimentary, Absorbent and Hemopoietic, Circulatory, Respiratory, Integumentary, Urinary, Reproductive, Nervous, and Locomotory. As an accurate diagnosis lies at the foundation of all successful treatment, so an intimate knowledge of the best methods of arriving at diagnostic accuracy is essential to the attainment of substantial renown by the physician. The author has grouped his facts with clearness and accuracy, making pleasant reading of even routine and dry portions of his book; he has given much valuable information in a succinct manner; his sentences are terse, yet rarely epigrammatic, and never dogmatic; and yet, withal, he is scientifically precise in description, data, and conclusions. A copious index, prepared with great care, adds much to the value of the work, and the publisher has contributed his full share to perfecting a handsome volume. Whoever failed to purchase the first issue of this manual should not omit to provide himself with this one.

---

**Physicians' Pocket Day-Book and Ledger Combined.** By DR. S. L. KILMER, South Bend, Ind.

During the past few years several forms of combination account books for physicians have been devised, their object being to relieve the hurried physician from the irksomeness of ordinary book-keeping. Dr. Kilmer's book appears to be *practically* adapted to the purpose indicated, and we propose to test it by a personal trial. We have not the space for a full description of the system inaugurated by Dr. Kilmer, but anyone can easily satisfy himself on the subject by addressing the inventor as above, who will send descriptive circulars with complete information.

---

**The Life Insurance Examiner—A Practical Treatise upon Medical Examinations for Life Insurance.** By CHARLES F. STILLMAN, M. S., M. D., Medical Examiner for the Mutual Life Insurance Company; Clinical Professor of Orthopedic Surgery in the Woman's Medical College of the New York Infirmary, etc., etc. 8vo, pp. 188, 16, viii. New York: The Spectator Company, 16 Dey street. 1888.

The growing importance of life insurance in the economics of society merits the most careful consideration of every problem connected therewith. The medical department is, perforce, the most important administrative bureau of any insurance company that takes risk on life or health. The Medical Director must surround himself with honest, capable, educated examiners, with whom he must establish and maintain relations of confidence, mutual respect, and even personal friendship. Most life insurance companies, certainly all good ones, are willing to pay adequate compensation to their medical examiners, and, in return, have a right to expect prompt, faithful, and impartial reports as to the physical condition of applicants. Dr. Stillman has, in his book, given explicit directions as to the methods of properly performing these duties, together with many useful facts grouped in convenient form for ready reference, that are of importance, and must be known to the successful medical examiner. It is a book that ought to be in the hands of every life insurance medical officer, and we think it would pay the companies in the end to present their examiners each with a copy.

---

**The Vest-Pocket Anatomist:** Founded upon Gray, containing Dissection Hints and Visceral Anatomy. By C. HENRI LEONARD, A. M., M. D., Professor of Medical and Surgical Diseases of Women, and Clinical Gynecology, Detroit College of Medicine, etc., etc. 12mo, pp. 304, 193 illustrations. Price, $1. Cloth. Detroit: The Illustrated Medical Journal Co.

This little volume has shown its popularity by passing through fourteen editions, and at this late day does not need an extended notice. The brain and its membranes, the eye, ear, throat, and the viscera of the generative organs, male and female, forms the new subject-matter of this edition. It is one of the best dissection manuals that we know of. The type is clear and distinct, and the illustrations are fully equal to those in "Gray," upon which work it is founded. We notice, too, that it has passed through many editions in England, where dissection manuals are plenty, which shows that the author has a most practical way of arranging the facts of the subject in a form easily carried, and yet sufficient for most purposes. It should be in the hands of every student, and it is also most convenient for reference for every physician, as he needs to refresh his memory on that all-important, though often easily forgotten, subject of anatomy.

---

**A Manual of Dietetics for Physicians, Mothers and Nurses.** By W. B. Pritchard, M. D., New York City. 12mo, pp. 88. Price, fifty cents. New York: Published by the Dietetic Publishing Co., 115 Fulton street.

This book is a compilation, in convenient form, of many important facts that the persons for whom it is especially written ought to know. It is a book that could find a useful place in every household, and its price places it within the reach of almost every one who has any need of it.

